# Ensemble learning *via* supervision augmentation for white matter hyperintensity segmentation

**DOI:** 10.3389/fnins.2022.946343

**Published:** 2022-09-15

**Authors:** Xutao Guo, Chenfei Ye, Yanwu Yang, Li Zhang, Li Liang, Shang Lu, Haiyan Lv, Chunjie Guo, Ting Ma

**Affiliations:** ^1^School of Electronics and Information Engineering, Harbin Institute of Technology, Shenzhen, China; ^2^Peng Cheng Laboratory, Shenzhen, China; ^3^International Research Institute for Artifcial Intelligence, Harbin Institute of Technology, Shenzhen, China; ^4^Department of Radiology, The First Hospital of Jilin University, Changchun, China; ^5^Mindsgo Life Science Company, Shenzhen, China

**Keywords:** supervision augmentation, ensemble learning, uncertainty, deep learning, white matter hyperintensity segmentation

## Abstract

Since the ambiguous boundary of the lesion and inter-observer variability, white matter hyperintensity segmentation annotations are inherently noisy and uncertain. On the other hand, the high capacity of deep neural networks (DNN) enables them to overfit labels with noise and uncertainty, which may lead to biased models with weak generalization ability. This challenge has been addressed by leveraging multiple annotations per image. However, multiple annotations are often not available in a real-world scenario. To mitigate the issue, this paper proposes a supervision augmentation method (SA) and combines it with ensemble learning (SA-EN) to improve the generalization ability of the model. SA can obtain diverse supervision information by estimating the uncertainty of annotation in a real-world scenario that per image have only one ambiguous annotation. Then different base learners in EN are trained with diverse supervision information. The experimental results on two white matter hyperintensity segmentation datasets demonstrate that SA-EN gets the optimal accuracy compared with other state-of-the-art ensemble methods. SA-EN is more effective on small datasets, which is more suitable for medical image segmentation with few annotations. A quantitative study is presented to show the effect of ensemble size and the effectiveness of the ensemble model. Furthermore, SA-EN can capture two types of uncertainty, aleatoric uncertainty modeled in SA and epistemic uncertainty modeled in EN.

## 1. Introduction

White matter hyperintensities (WMHs), defined as hyperintensities on T2-weighted (T2w) or T2-weighted fluid-attenuated inversion recovery (T2-FLAIR) magnetic resonance (MR) images, are located in cerebral white matter tissues and of varying sizes (Wardlaw et al., 2013; Liang et al., 2021). These abnormal signals mainly come from normal aging and a lot of neuropsychiatric disorders, such as dementia and small-vessel diseases (Wallin et al., 2018). Based on the quantitative analysis of WMHs, many studies have shown that the quantitative characterization of WMHs plays an important role in various clinical studies of nervous system diseases (Brickman et al., 2018; Dadar et al., 2019). However, manually labeling lesions is a time-consuming process, and human error is unavoidable. Therefore, automatic MRI segmentation of WMHs has important potential for clinical applications.

**Figure 1:**
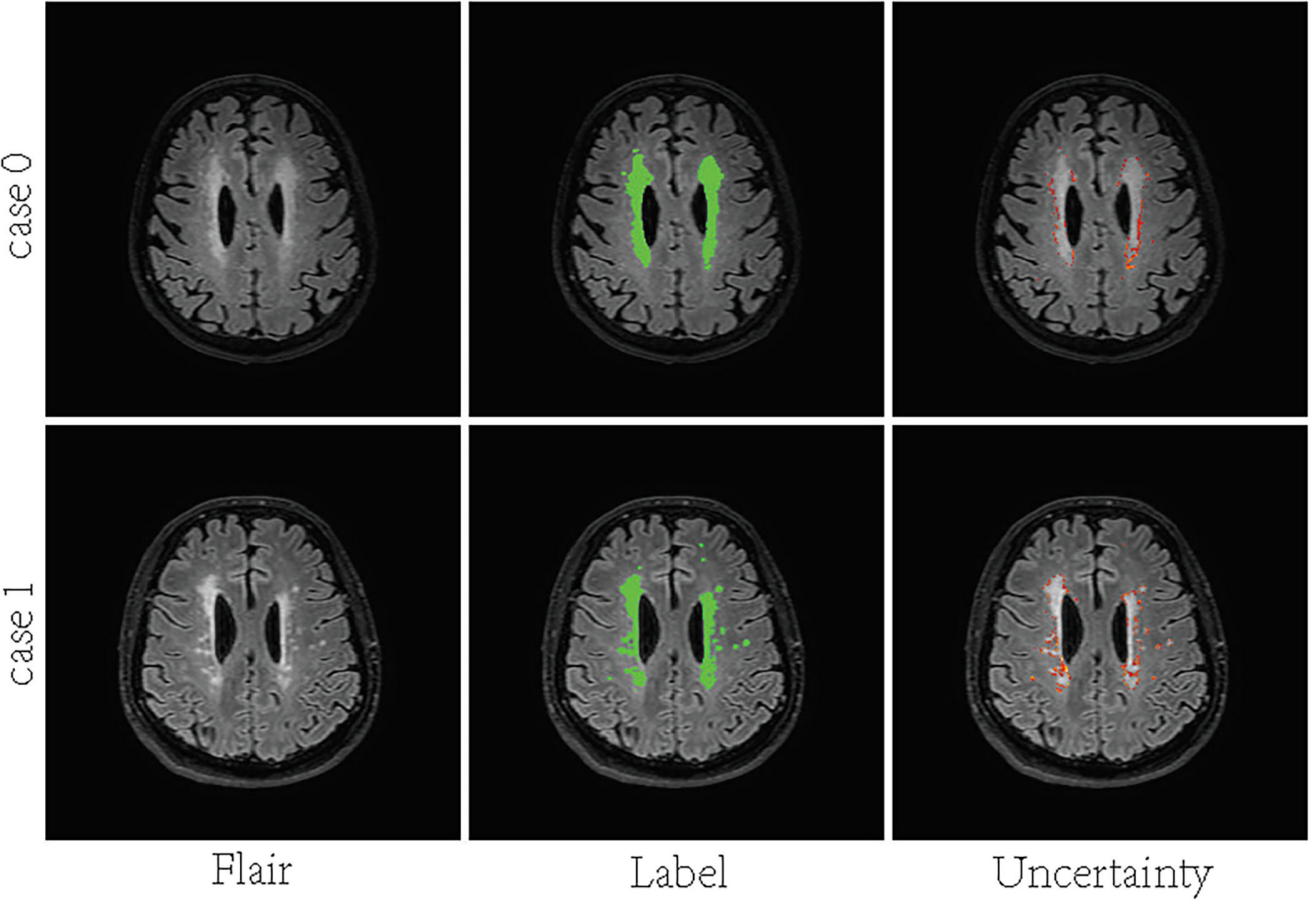
Visualization of uncertainty maps on two training slices computed by Bayesian U-Net. From left to right: Flair MR images, the associated ground truth, and the associated uncertainty map.

In recent years, deep convolutional neural networks (DCNN) have achieved state-of-the-art performance in medical image segmentation (Litjens et al., 2017; Hu et al., 2018). One of the fundamental facts contributing to such success is the massive training data with reliable annotations. However, medical image segmentation annotations are inherently noisy and uncertain due to the ambiguous boundary of the lesion and inter-observer variability. For White matter hyperintensities, the lesions near the ventricles are more prominent and the boundaries look sharper. Deeper regions tend to have blurrier boundaries. As shown in Figure 1, high uncertainty is mainly distributed in deeper regions, especially the lesion boundary and some smaller lesions. The manual segmentation was highly reliable in the region close to the ventricle, even at the border. Since the high capacity of deep neural networks (DNN), the DL-based approaches are easy to overfit to labels with noise and uncertainty, which may lead to biased models with weak generalization ability (Lee et al., 2016; Kohl et al., 2018; Zhang et al., 2021). Many methods overcome this challenge by leveraging multiple annotations per image (Hu et al., 2019; Mirikharaji et al., 2021). When facing an uncertain situation in practice, humans also tend to produce multiple plausible assumptions. Similarly, images can be better evaluated using annotations from a group of annotators. The advantage of multiple annotations is that they can provide diverse supervision information during model training (Yang and Xu, 2020; Mirikharaji et al., 2021). However, in a common real-world scenario, per image have only one ambiguous and noisy annotation per image.

One natural question raised from the above analysis could be can we obtain diverse supervision information based on a single annotation? Training set biases can sometimes be addressed with the dataset resampling (Chawla et al., 2002; Ren et al., 2018), i.e., choosing the correct proportion of labels to train a network on, or more generally by assigning a weight to each example and minimizing a weighted training loss (Freund and Schapire, 1997; Chang et al., 2017). The semantic segmentation task entails assigning pixel-wise class labels to the entire image. The annotations of different pixels contain varying degrees of ambiguity and noise. For example, the annotation of pixels at the boundary of the lesion has high noise and ambiguity (Lakshminarayanan et al., 2017; Beluch et al., 2018). However, the existing deep learning models usually lack the consideration of annotation reliability at the pixel level when calculating the loss function, such as the commonly used cross-entropy or dice loss. In the above regard, this paper proposes a supervision augmentation (SA) method to obtain diverse supervision information to achieve similar effects of multiple annotations instead of directly obtaining multiple annotations. Concretely, Bayesian CNN is used to estimate the uncertainty of annotation. When calculating the loss function, some pixels with high uncertainty will be filtered out. By changing the filtering threshold, diverse supervision information can be obtained when calculating the loss function. Then, SA is combined with ensemble learning (SA-EN) for White Matter Segmentation. Different base models train with different supervision information in EN. The experimental results on two white matter hyperintensity segmentation datasets demonstrate that SA-EN can get the optimal accuracy compared with other state-of-the-art ensemble methods. SA-EN is more effective on small datasets. This is more suitable for medical image segmentation with few annotations. Furthermore, the aleatoric uncertainty (Kendall and Gal, 2017) can be modeled in SA. The epistemic uncertainty (Kendall and Gal, 2017) can be modeled in EN.

The main contributions of this article can be summarized as follows: (1) This paper proposed a SA method, which can obtain diverse supervision information for common single-label scenarios without adding additional data labeling burden. (2) The experiments show EN *via* supervision augmentation (SA-EN) outperforms the state-of-the-art methods in white matter hyperintensity segmentation. (3) SA-EN is more effective on small datasets. (4) SA-EN can capture two types of uncertainty, aleatoric uncertainty modeled in SA and epistemic uncertainty modeled in EN. The notations we use throughout the paper are summarized in Table 1.

**Table 1:** Notations used in the paper.

**Notation**	**Description**
λ	Uncertainty threshold.
*W*	Network's weights.
*p*(*W*|*X, Y*)	W's posterior distribution over the training sets.
*x*_*n*_, *y*_*n*_	Training Images and corresponding annotations.
$\tilde{x},\;\tilde{y}$	Testing image and corresponding predictive label.
*U*(*x*)	The uncertainty map of the training sample *x*.
*Mask*(*x*_*m*_)	Binary mask to indicate whether the current pixel is involved in the loss function.
*L*	Loss function.
*l*	The label class.
*K*	Number of base model.
*f*_*k*_, *p*_*k*_	The *k*_*th*_ base model in the ensemble learning and corresponding prediction probability.
θ	Dropout ratio.
*T*	The number of dropout samplings.
*Mean, Var*	Mean probability, mean probability variation.

## 2. Related works

### 2.1. Medical image segmentation

In recent years, deep learning has made great development in medical image segmentation. The U-Net (Ronneberger et al., 2015) is one of the most commonly used convolutional network structures in medical image segmentation. By adopting an encoder-decoder network structure and skip connection, it can combine features of the different decoding layers with features of the different coding layers (Drozdzal et al., 2016; He et al., 2016). Some later works also achieve higher performance by improving the architecture of U-Net, such as AttU-Net (Oktay et al., 2018) and U-Net++ (Zhou et al., 2019). Oktay et al. (2018) introduced the attention mechanism (Vaswani et al., 2017) into U-Net, which can suppress irrelevant areas in the input image and highlight the salient features of specific local areas. Zhou et al. (2019) propose a new segmentation architecture based on nested and dense skip connections. This designed skip connection reduces the gap between the feature maps of the encoding and decoding sub-networks. Many DCNNs have been proposed in the literature for white matter hyperintensity segmentation. Moeskops et al. (2018) proposed a patch-based deep CNN to segment brain tissues and WMH in MR images. Guerrero et al. (2018) proposed a network called uResNet which combines the strengths of both U-Net and residual neural networks to segment hyperintensities. Li et al. (2018) proposed an ensemble of three U-Net's with different random weight initializations to automatically detect WMH. Sundaresan et al. (2021) propose an ensemble triplanar network that combines the predictions from three different planes of brain MR images to provide an accurate WMH segmentation.

### 2.2. Uncertainty

In machine learning, uncertainty has been classified into aleatoric and epistemic types. The aleatoric reflects the inherent noise in the data (Kendall and Gal, 2017). The epistemic uncertainty is associated with the network's parameters (Kendall and Gal, 2017). It has been shown in previous research (Pereyra et al., 2017) that the softmax output of a neural network tends to be overconfident. Moreover, the cross-entropy loss can interpret as a maximum likelihood estimation, which is not suited for the estimation of a predictive distribution's variance (Sensoy et al., 2018). Bayesian networks are an efficient method for modeling (epistemic) uncertainty (MacKay, 1992; Barber and Bishop, 1998). But their implementation is difficult and computationally expensive. Arguably, Monte Carlo dropout (MC-Dropout) (Gal and Ghahramani, 2016) is one of the most well-known techniques to quantify the model's uncertainty in deep learning methods. When dropout is applied at training and testing time, it can be used to perform a variational approximation of a Bayesian neural network that has Bernoulli distributions as prior. Deep ensembles are another sampling-based approach for the estimation of the predictive uncertainty of DNNs (Lakshminarayanan et al., 2017). Lakshminarayanan et al. (2017) and Beluch et al. (2018) have also shown deep ensembles often outperform Monte-Carlo dropout, even requiring significantly less samples. In medical image segmentation, many methods further improve the segmentation accuracy through uncertainty analysis. Hiasa et al. (2019b) use Bayesian U-Net for personalized musculoskeletal modeling. Yu et al. (2019) present a novel uncertainty-aware semi-supervised learning framework for left atrium segmentation from 3D MR images by additionally leveraging the unlabeled data. Tang et al. (2022) propose an uncertainty guided network referred to as UG-Net for automatic medical image segmentation.

### 2.3. Ensembles learning

Ensemble learning is a powerful machine learning paradigm that has exhibited apparent advantages in many applications (Zhou, 2021). By using multiple learners, the generalization ability of an ensemble can be much better than that of a single learner (Hansen and Salamon, 1990). Ensembles are widely used in machine learning (Dietterich, 2000), such as Adaboost (Schapire, 1990), Bagging (Breiman, 1996), Stacking (Wolpert, 1992), etc. Similarly, the network ensemble is a popular approach to improving the generalization of DL networks (Ganaie et al., 2021). Typically, the most network ensemble approach is the k-fold cross-validation strategy that trains multiple networks with different subsets of training data and random initialization of the networks (Krogh and Vedelsby, 1994; Li et al., 2018; Sundaresan et al., 2021). Many works also use different network structures to realize a network ensemble (Garipov et al., 2018; Herron et al., 2020; Chen et al., 2021). There are also methods of the implicit ensemble with dropout-like schemes (Srivastava et al., 2014; Huang et al., 2016). DL has large parameters, which are easy to converge to the local minimum, so it is also suitable for network ensembles. Li et al. (2018) proposed an ensemble of three U-Net's with different random weight initializations to automatically detect WMH. Li et al. (2022) present a pipeline using deep fully convolutional network and ensemble models, combining U-Net, SE-Net, and multi-scale features, to automatically segment WMHs and estimate their volumes and locations. Sundaresan et al. (2021) achieves ensemble by combining three different planes of brain MR images.

## 3. Methods

**Figure 2:**
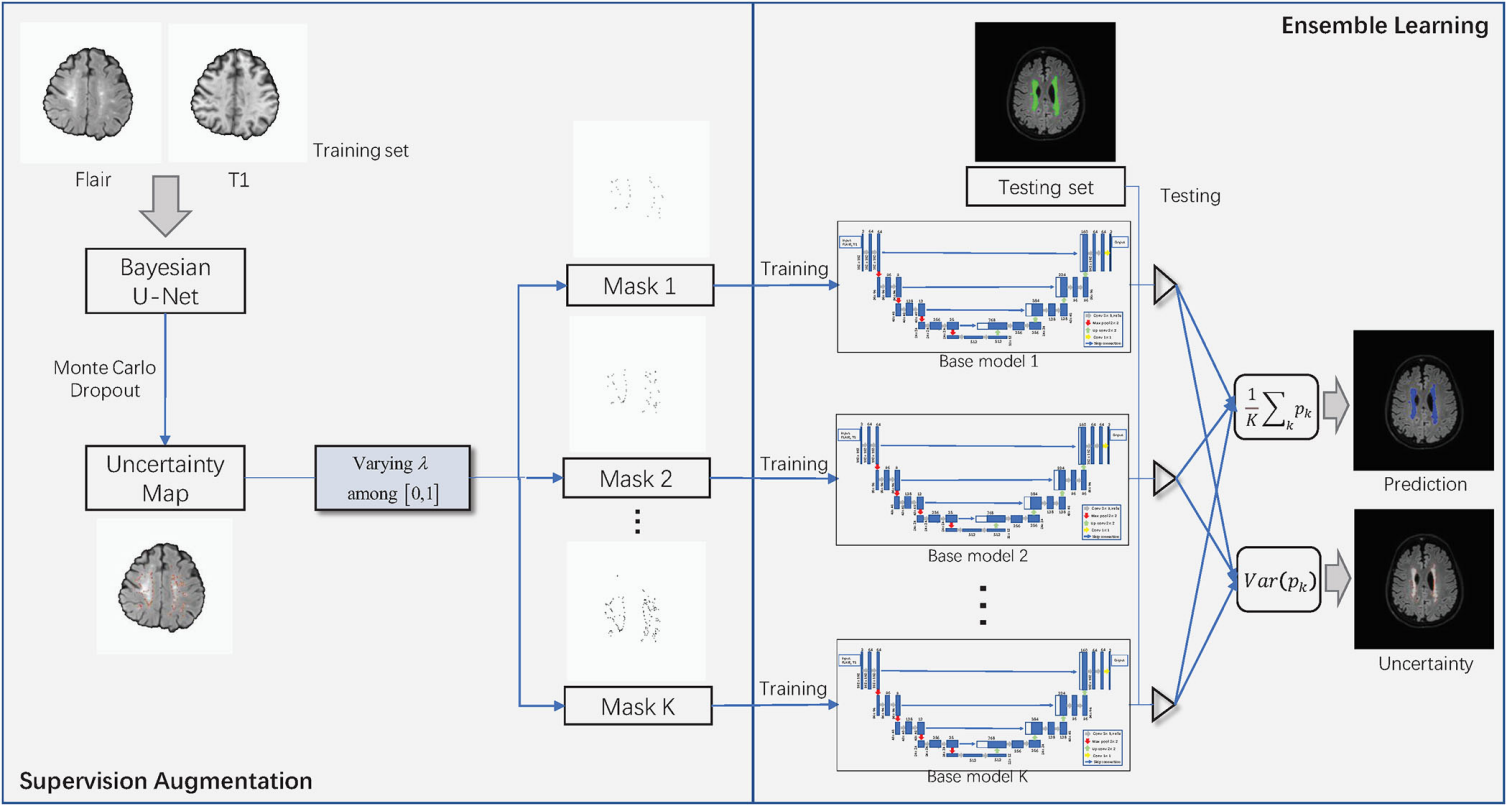
The overall structure of SA-EN. It consists of two parts: supervision augmentation and ensemble learning.

Figure 2 shows the overall structure of SA-EN, which consists of two parts: SA and EN. In the part of SA, Bayesian CNN with the U-Net architecture, using Monte Carlo dropout is used to estimate the uncertainty map of annotation. By changing the threshold λ of the filter, different binary masks can obtain for calculating the loss function. The generated binary mask indicates which pixels are involved in the loss function. In EN, different base models train with different masks. Finally, the trained base models are fused to obtain the final results. It extends traditional single-loss, single-output network structures to multiple outputs by SA and EN. As shown in Figure 2, SA-EN can get segmentation results and model uncertainty simultaneously.

### 3.1. Supervision augementation

**Figure 3:**
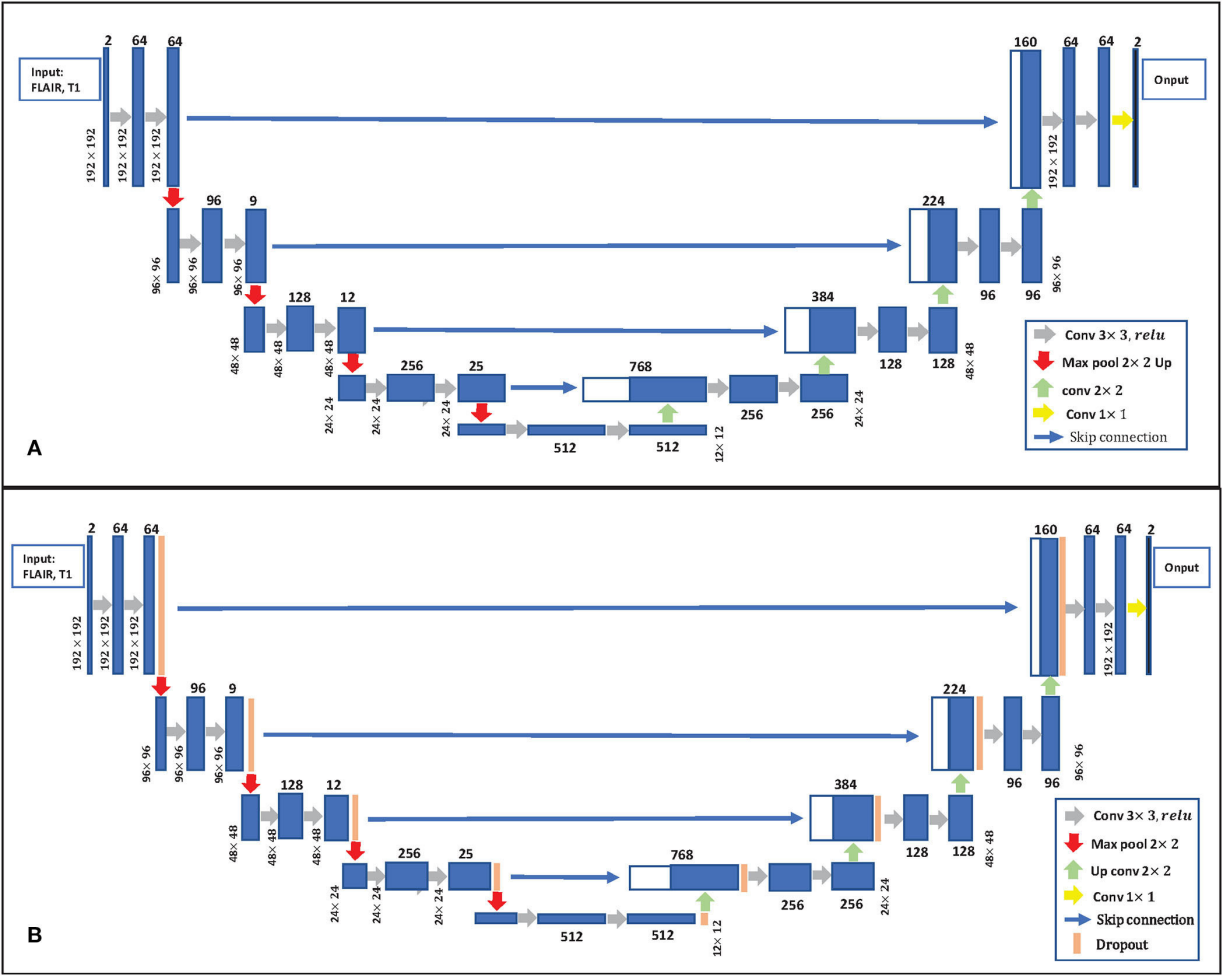
**(A)** 2D U-Net Architecture. **(B)** Bayesian U-Net. The U-Net inserts the dropout layer before each max pooling layer and after each up-convolution layer.

**Supervision augementation:** The segmentation model based on multi-annotation is more robust in reducing the effects of label noisy and ambiguity (Hu et al., 2019; Mirikharaji et al., 2021). The advantage of multiple annotations is that they can provide diverse supervision information during model training (Yang and Xu, 2020; Mirikharaji et al., 2021). Instead of directly acquiring multiple annotations, this paper proposes a SA method to obtain diverse supervision information to achieve similar effects of multiple annotations. With the guidance of the estimated annotation uncertainty, supervision augmentation filter out some pixels with unreliable annotations and preserves only the reliable ones (low uncertainty) when calculating the loss function. As shown in Figure 2, Bayesian convolutional neural networks are used to estimate the uncertainty map of annotation. The annotation of pixels with high uncertainty usually has large ambiguity and is noisy (Lakshminarayanan et al., 2017; Beluch et al., 2018). Then, we can set an uncertainty threshold λ. Accordingly, one binary mask can be made by comparing the uncertainty with λ. For example, when the uncertainty of one pixel is greater than λ, the mask corresponding to this pixel is set to 0 and will not be involved in the loss function. By setting different thresholds λ, multiple binary masks can obtain on a single annotation. Different supervision information can obtain by calculating loss functions using different binary masks. Last, different base models train by different masks in EN. This paper uses Bayesian convolutional neural networks with the U-Net architecture, using Monte Carlo dropout to estimate the uncertainty map of annotation. The details are as follows:

**Uncertainty estimation:** Gal and Ghahramani (2016) developed a new theoretical framework casting dropout training in DNNs as approximate Bayesian inference in deep Gaussian processes. This paper follows Gal and Ghahramani (2016) to estimate uncertainty, which used the dropout at the inference phase. The details of Bayesian U-Net are shown in Figure 3B. Bayesian U-Net allows the computation of epistemic uncertainties by modeling a posterior distribution *p*(*W*|*X, Y*) over the network's weights *W*. Suppose we have a training data set of images *X* = {*x*_*n*_} and their labels *Y* = {*y*_*n*_}, *n* = 1, 2, ...*N*. In traditional deep learning, the predictive label ỹ of a testing image $\tilde{x}$ can be expressed as $p(\tilde{y}|\tilde{x})=Softmax[f(\tilde{x};W)]$. The Bayesian neural network is given by the marginalization of *W* as:


(1) $$p(\tilde{y}=l|\tilde{x},X,Y)=\int p(\tilde{y}=l|\tilde{x},W)p(W|X,Y)dW$$

where ỹ is the output label of a pixel, *l* is the label class, and *p*(*W*|*X, Y*) is the posterior distribution. However, finding the exact posterior is intractable, but an approximation *q*(*W*) can be obtained using variational inference, by minimizing the Kullback-Leibler (KL) divergence $KL\left[q\left(\tilde{W}\right)||p\left(\tilde{W}|X,Y\right)\right]$. Gal and Ghahramani (2016) proved that approximation of the posterior distribution is equivalent to the dropout masked distribution *q*(Ŵ), where Ŵ = *W*  ·  *diag*(*z*) and *z*~*Bernoulli*(*θ*), and *θ* is the dropout ratio. Then, Equation (1) can be approximated as


(2) $$\begin{array}{c} \begin{array}{l} p(\tilde{y}=c|\tilde{x},X,Y)\approx \int p(\tilde{y}=l|\tilde{x},\;\tilde{W})q(\tilde{W})d\tilde{W}\\ \;\approx \frac{1}{T}\sum_{t=1}^{T}Softmax[f(\tilde{x},\;\tilde{W})] \end{array} \end{array}$$

where *T* is the number of dropout samplings. Dropout is used at test time to retrieve multiple Monte Carlo (MC) samples by processing the input $\tilde{x}$, *T* times. This paper use probability variation as uncertainty, given as follows.

Mean probability: For each pixel on a training sample, a mean probability was calculated from the *T* pixel-level probability maps.


(3) $$Mean(\tilde{y}=c|\tilde{x},X,Y)\approx \frac{1}{T}\sum_{t=1}^{T}Softmax[f(\tilde{x},\tilde{W})]$$

Mean probability variation: For each pixel on a training sample, probability variation was defined as the SD of the *T* pixel-level probability maps. If the model is certain, the measure should be close to 0.


(4) $$\begin{array}{l} Var(\tilde{y}=c|\tilde{x},X,Y)\\ \;\approx \frac{1}{T}\sum_{t=1}^{T}Softmax{\left[f(\tilde{x},\tilde{W})\right]}^{T}Softmax\left[f(\tilde{x},\tilde{W})\right]\\ \;-p{\left(\tilde{y},\tilde{x},X,Y\right)}^{T}p(\tilde{y},\tilde{x},X,Y) \end{array}$$

**Figure 4:**
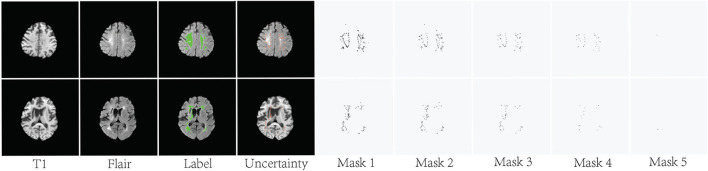
Visualization of the uncertainty maps and generated binary masks on two training slices. The green areas indicate ground truth.

As shown in Figure 4, the pixels with higher uncertainty are mainly distributed at the lesion boundary and contain more ambiguity and noise. *U*(*x*) represents the uncertainty map of the training sample *x* and is obtained by normalizing the result of Formula 5. The binary mask *Mask*(*x*_*m*_) is used to indicate whether the current pixel is involved in the loss function. *m* represents the index of the pixel in the current image.


(5) $$Mask(x_{m})=\left\{ \begin{array}{l} 1,\;U(x_{m})\leq \lambda \\ 0,\;U(x_{m})>\lambda \end{array}\right.$$

λ is the uncertainty threshold. If the uncertainty of one pixel *x*_*m*_ is greater than λ, *Mask*(*x*_*m*_) is set to 0 and is not involved in the loss function. Figure 4 shows some examples of the generated binary masks.

### 3.2. Ensembles learning *via* supervision augementation

In contrast to ordinary machine learning approaches, which learn one hypothesis from training data, ensemble methods try to construct a set of hypotheses and combine them to use (Hansen and Salamon, 1990; Zhou, 2021). The generalization ability of EN can be much better than that of a single learner. It is helpful to reduce the over-fitting problems of a complex model on the training data. This paper proposes to addresses the automated white matter hyperintensity segmentation problem by an ensemble approach to combine several models with the same architecture. Different from the previous methods, this work uses diverse supervision information to train different base learners. The following experiments will show the effectiveness of the ensemble model *via* qualitative and quantitative analysis.

The intention to use ensemble models includes two aspects: 1) networks trained with different supervision information can learn different attributes of the training data, thus the ensemble of them could boost the segmentation results. 2) bias-variance trade-off (Bauer and Kohavi, 1999; Zhou et al., 2002). Bias and variance are critical for determining the behavior of prediction models and understanding the occurrence of overfitting and underfitting. This work aims to lower the model variance by averaging the model output. Deep learning with millions of parameters and overtrained on different boot-strapped/subsampled training sets can qualify for unbiased and highly variant models. The following experiments will quantitatively analyze that the ensemble model served as the typical bias-variance trade-off.


(6) $$L=-\frac{1}{N}\frac{1}{M}\sum_{n}\sum_{m}\sum_{c=1}^{C}Mask(x_{n,m})y_{n,m}log(p_{n,m}^{c})$$

As shown in Figure 2, *K* U-Net models with the same architecture are trained with different supervision information. Formula 7 is the loss function used in base model training. It introduces a binary mask in the cross-entropy loss function. *N* and *M* represent the number of samples and the number of pixels, respectively. *C* represents the number of categories. This training creates sufficient diversity in the trained base models *f*_*k*_ to allow the averaged predictions of the ensemble to outperform the individual models significantly. Each trained base model will predict the test image and generate a probability map *p*_*k*_. Then, the resulting *K* probability maps will be averaged $\frac{1}{K}\sum_{k=1}^{K}p_{k}$. Finally, the averaged map is transformed into the segmentation result. Referring to Formula 5, we can also estimate the model uncertainty according to these probability maps *p*_*k*_. The details of the algorithm are given in Algorithm 1.

**Algorithm 1:** **Ensembles learning *via* Supervision Augementation**.

**Intput:** Data set $D=\left(x_{1},y_{1}\right),\left(x_{2},y_{2}\right),...,\left(x_{n},y_{n}\right)$;
Loss function L;
Number of learning rounds *K*.
**Process:**
for k = 1,...,K:
Select one value from [0-1] as λ; % Generate different binary mask *Mask*_λ_
*f*_*k*_ = $L\left(D,Mask_{\lambda }\right)$ % Train a base model *f*_*k*_ by applying the different binary mask.
end.
**Output:** *F*(*x*) = $argmax\left\{\frac{1}{K}\overset{K}{\underset{k=1}{\sum }}p_{k}\right\}$; % Segmentation results
*U*(*x*) = *Var*(*p*_*k*_). % Uncertainty estimation

## 4. Experiments

### 4.1. Datasets

**MICCAI dataset:** The dataset is provided by the WMH segmentation challenge in MICCAI 2017 (Kuijf et al., 2019). It consists of 60 cases of brain MRI images (3D T1-weighted image and 2D multi-slice FLAIR image) with manual annotations of white matter hyperintensity (binary masks) from three different institutes/scanners. The manual reference standard is defined on the FLAIR image. So, a 2D multi-slice version of the T1 image was generated by re-sampling the 3D T1-weighted image to match with the FLAIR one. In this paper, all cases are randomly assigned into 5-folds. Then, the 5-folds are randomly assigned into a training set (4-fold) and a testing set (1-fold).

**Local dataset:** The local dataset is retrospectively collected by the First Hospital of Jilin University, China. All the MRI data were collected with the approval of the local ethics committee. It consists of 300 cases of brain MRI images (3D T1-weighted image and 3D FLAIR image) with manual annotations of white matter hyperintensity (binary masks). The subjects were between the ages of 33 and 87 (including 129 males and 171 females). For this study, the dataset was designated as a local dataset for simplicity. All patients are randomly assigned into 5-folds. Then, the 5-folds are randomly assigned into a training set (4-fold) and a testing set (1-fold).

### 4.2. Implementation details

This paper performed intra-subject coregistration between Flair and T1 using FSL FLIRT affine transformation (Jenkinson and Smith, 2001; Jenkinson et al., 2002). After coregistration, global inhomogeneity corrections of T1 and Flair were performed by advanced normalization tools (ANTs) (Tustison et al., 2010). Gaussian normalization was employed to normalize the voxel intensities of each subject with a mean zero and a standard deviation of one. In this study, all the networks train using Pytorch using NVIDIA TESLA V-100 (Pascal) GPUs with 32 GB memory. This paper adopts the architecture of a fully convolutional network 2D U-Net (Ronneberger et al., 2015) initialized by a random Gaussian distribution, as shown in the Appendix. We optimized all configurations with the Adam optimizer with the learning rate 1e-4 and the weight decay 1e-5 (Kingma and Ba, 2014). The batch size is set to 16. For the MICCAI dataset and local dataset, images and annotation labels were randomly cropped to 128 × 192 patches and 192 × 192 patches, respectively. At the inference stage, the segmentation probability maps and label maps were predicted by the sliding windows technique with 50% overlaps. The standard data augmentation techniques are used to avoid overfitting (Li et al., 2018), including randomly flipping, randomly rotating, and randomly mirroring. To ensure the experiment's objectivity, this paper strictly guarantees that the training parameters of comparative experiment are consistent. Four metrics were used to evaluate the performance of different methods based on the segmentation results: (1) Dice, (2) a modified Hausdorff distance (95th percentile; H95), (3) recall: the ratio of true positives from each method to the manually traced WMHs, and (4) F1-score (F1).

### 4.3. Results

#### 4.3.1. Comparison of segmentation accuracy

**Figure 5:**
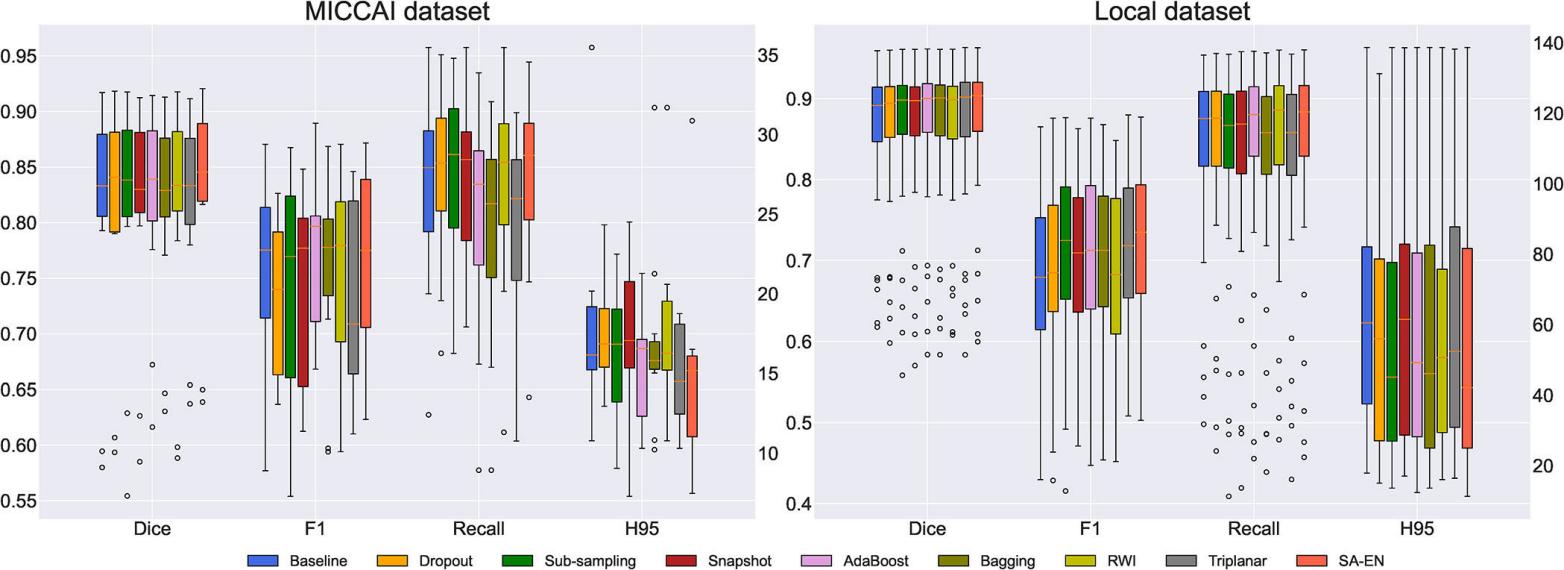
Box plots of different methods in terms of Dice, F1, Recall, and H95. The boxplots show the median and the 25 and 75% percentiles of the metrics distribution.

We compare the proposed method with prior ensemble methods, including Dropout (Srivastava et al., 2014; Huang et al., 2016), Sub-sampling (Krogh and Vedelsby, 1994), Snapshot (Gao et al., 2017), AdaBoost (Schapire, 1990), and Bagging (Breiman, 1996). Sub-sampling strategy trains multiple base networks with different subsets of training data. In this paper, we randomly divide the training set into five subsets to train the base network. Table 2 shows the quantitative results of different methods on the MICCAI dataset and local dataset segmentation. For a fair comparison, the base models used in various ensemble methods adopt the U-Net model with the same structure. All ensemble methods use five base models. The base model structure of U-Net is described in the Appendix, as shown in Figure 3A. All ensemble methods use the same fusion method. First, the resulting probability maps obtained from different base models are averaged. Then, the averaged map is transformed into the segmentation result. It can be seen from the Table 2 that the EN method can effectively improve the performance of baseline U-Net. SA-EN achieves a significant dice gain over baseline U-Net for the MICCAI dataset (↑1.98) and the local dataset (↑1.08) segmentation. SA-EN gets the optimal accuracy compared with other state-of-the-art ensemble methods (Bagging, AdaBoost, and Snapshot). Figure 5 also shows the four evaluation metrics (Dice, F1, Recall, and H95) of the different methods. Table 3 shows the quantitative results of the four evaluation metrics (Dice, F1, Recall, and H95). The *p* < 0.05 in Table 3 also proves that the difference is statistically significant. Especially on the Local dataset, SA-EN has the best performance on all four metrics. After checking the original slices and segmentation results, the outliers (hard examples) in Figure 5 contain relatively many small lesions and fuzzy slices. Small lesions have always been a difficulty of segmentation, and the corresponding segmentation accuracy is low. Fuzzy slices will also lead to low segmentation accuracy.

**Table 2:** Performance (Dice, %, higher is better) of different methods on two datasets.

**Task**	**Method**	**Base model 1**	**Base model 2**	**Base model 3**	**Base model 4**	**Base model 5**	**Ensemble**
MICCAI dataset	Baseline	✗	✗	✗	✗	✗	80.60
	Dropout	✗	✗	✗	✗	✗	80.92
	Sub-sampling	79.85	79.82	79.95	79.71	79.50	81.21
	Snapshot	80.63	80.39	80.40	80.65	80.34	81.02
	AdaBoost	80.59	80.99	80.88	81.02	80.90	81.73
	Bagging	80.34	79.76	79.69	80.51	80.71	81.93
	SA-EN	80.82	81.33	81.18	80.56	81.27	82.58
Local dataset	Baseline	✗	✗	✗	✗	✗	86.43
	Dropout	✗	✗	✗	✗	✗	86.61
	Sub-sampling	86.43	86.36	86.37	86.33	86.25	86.88
	Snapshot	86.19	86.29	86.37	86.47	86.38	86.72
	AdaBoost	86.43	86.45	86.56	86.54	86.61	87.05
	Bagging	85.82	85.63	85.75	85.72	85.74	86.80
	SA-EN	86.51	86.43	86.57	86.44	86.33	87.51

Note that “Baseline” represents the original U-Net. “Base model *k*” represents different base models in EN, and “Ensemble” represents the trained base models that are fused.

**Table 3:** Performance (Dice, F1, Recall, and H95) of different methods on two datasets.

	**MICCAI dataset**								**Local dataset**							
**Methods**	**Dice↑**	***p*** **-value**	**F1↑**	***p*** **-value**	**Recall↑**	***p*** **-value**	**H95↑**	***p*** **-value**	**Dice↑**	***p*** **-value**	**F1↑**	***p*** **-value**	**Recall↑**	***p*** **-value**	**H95↑**	***p*** **-value**
Baseline	80.60	<0.001	75.40	0.003	82.91	0.252	17.67	0.003	86.43	<0.001	67.81	<0.001	84.78	0.576	64.59	0.003
Dropout	80.92	0.049	73.04	0.024	84.10	0.282	17.19	0.035	86.61	<0.001	68.83	<0.001	84.59	0.123	56.61	0.026
Sub-sampling	81.21	0.061	74.05	0.120	84.23	<0.001	16.45	0.112	86.88	<0.001	71.13	<0.001	83.58	<0.001	54.89	0.344
Snapshot	81.02	<0.001	74.38	<0.001	83.57	0.069	17.43	0.005	86.72	<0.001	69.88	<0.001	83.65	<0.001	60.89	<0.001
AdaBoost	81.73	0.055	77.31	0.210	80.03	0.049	15.39	0.199	87.05	<0.001	70.67	<0.001	84.79	0.314	55.03	0.031
Bagging	81.93	0.046	75.51	0.127	79.37	0.049	16.85	0.089	86.80	<0.001	70.52	<0.001	83.31	<0.001	55.23	0.075
RWI	80.91	0.014	75.46	0.407	83.35	0.688	17.55	0.040	86.78	<0.001	68.39	<0.001	85.08	0.167	56.16	0.596
nar	81.29	0.008	73.37	0.032	79.48	<0.001	14.83	0.971	87.20	0.016	71.59	0.071	83.32	<0.001	60.17	0.015
SA-EN	82.58	✗	76.66	✗	83.52	✗	14.87	✗	87.51	✗	72.44	✗	84.90	✗	53.43	✗

Statistical analysis (*p*-value) of SA-EN compared with other CNN-based methods. Note that “Baseline” represents the U-Net.

**Figure 6:**
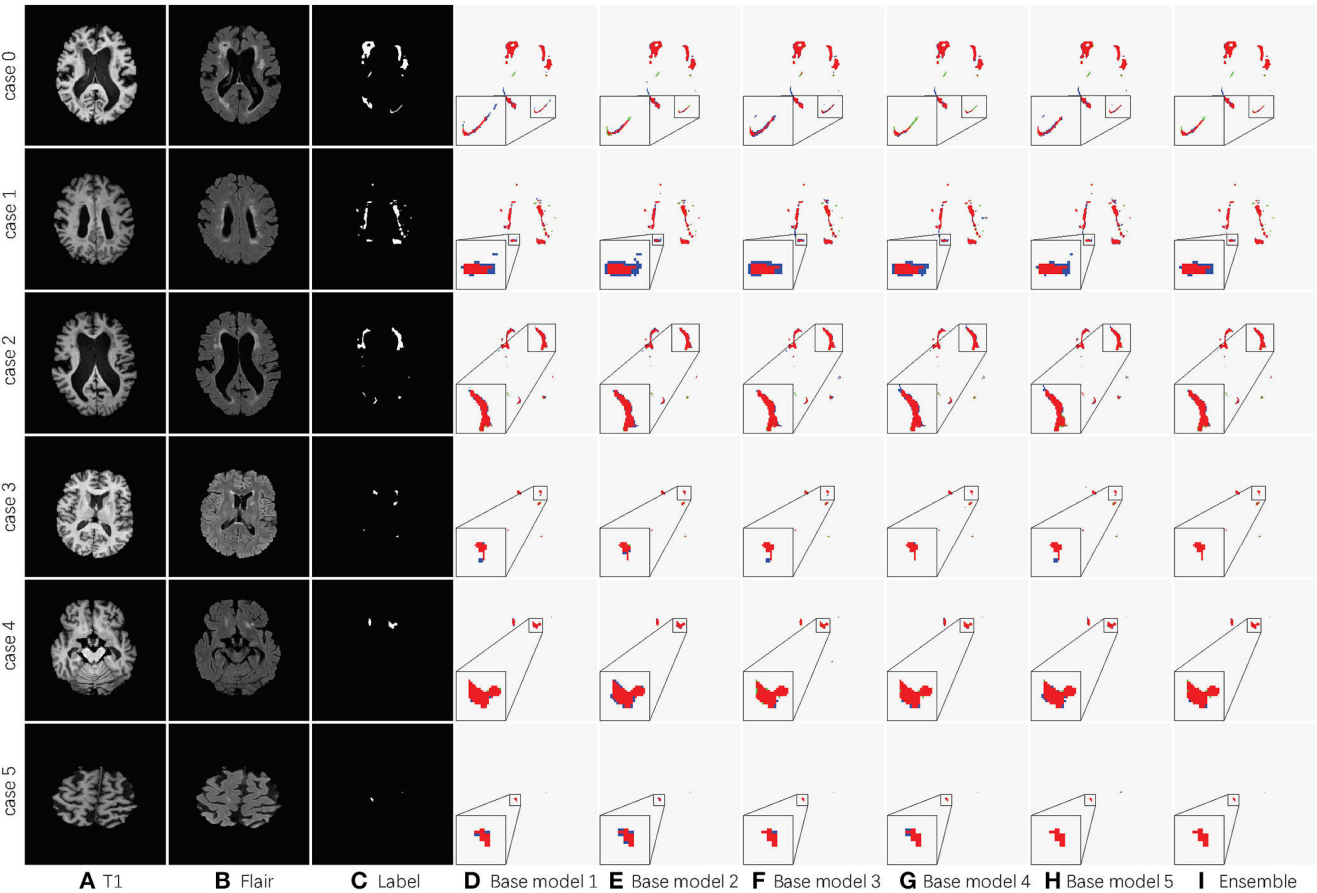
Detailed segmentation results of five base models and the ensemble. **(A)** T1 image; **(B)** Flair image; **(C)** Label; **(D)** Base model 1; **(E)** Base model 2; **(F)** Base model 3; **(G)** Base model 4; **(H)** Base model 5; **(I)** Ensemble. The red area in columns Base model 1, Base model 2, Base model 3, Base model 4, Base model 5, and Ensemble is the overlap between the segmentation result and label. The blue ones are the prediction errors. For better visualization, the regions inside the smaller yellow bounding box are zoomed into the larger bounding box.

Table 2 shows the accuracy of an ensemble is much stronger than base learners. For example, the base models of SA-EN on the local dataset have similar segmentation accuracy. However, the performance after the ensemble has been effectively improved. Although the segmentation accuracy of these base models is similar, the base models are diverse due to different supervision information. Figure 6 shows six cases segmented by five base models and their ensemble. The five base models generated significantly different results on the boundary. The model ensemble avoided the worst segmentation result.

Furthermore, we compare SA-EN with prior ensemble methods for white matter hyperintensity segmentation, including RWI (Li et al., 2018) and Triplanar (Sundaresan et al., 2021). RWI (Li et al., 2018) combines multiple U-Nets with different random weight initializations. Triplanar (Sundaresan et al., 2021) achieves ensemble by combining three different planes of brain MR images. Table 3 shows that SA-EN outperforms RWI and Triplanar on three metrics (Dice, F1, and Recall) on MICCAI datasets. SA-EN outperforms RWI and Triplanar on three metrics (Dice, F1, and H95) on Local datasets. Compared with SA-EN, the base model of the Triplanar ensemble method is limited to a maximum of 3. The value of *P* < 0.05 in Table 3 also proves that the difference is statistically significant.

#### 4.3.2. Performance on small and large lesions

We also analyzed the performance of SA-EN on large and small lesions, respectively. For each subject, the recall will be computed separately for individual lesions smaller than or equal to the median lesion size and for lesions larger than the median lesion size. The median size of lesions for Local and MICCAI datasets was 96 and 133, respectively. Table 4 shows that SA-EN significantly improves the segmentation accuracy of large lesions on the two datasets but has little effect on small lesions. We think that the differences between different masks generated by SA are mainly pixels with high uncertainty. As shown in Figure 1, a large number of pixels with high uncertainty are distributed on the edges of large lesions. Therefore, this may lead to the improvement of our proposed method for large lesions. The *P* < 0.05 in Table 4 also proves that the difference is statistically significant.

**Table 4:** Performance (Recall) on small and large lesions, respectively.

	**MICCAI dataset**				**Local dataset**			
	**Large lesion**		**Small lesion**		**Large lesion**		**Small lesion**	
**Methods**	**Recall**	***p*** **-value**	**Recal**	***p*** **-value**	**Recal**	***p*** **-value**	**Recal**	***p*** **-value**
Baseline	85.26	0.001	62.78	0.389	81.73	<0.001	52.56	0.223
SA-EN	86.83	✗	62.67	✗	82.01	✗	52.74	✗

Statistical analysis (*p*-value) of SA-EN compared with baseline. Note that “Baseline” represents the U-Net.

#### 4.3.3. Effect of sample size on model performance

**Figure 7:**
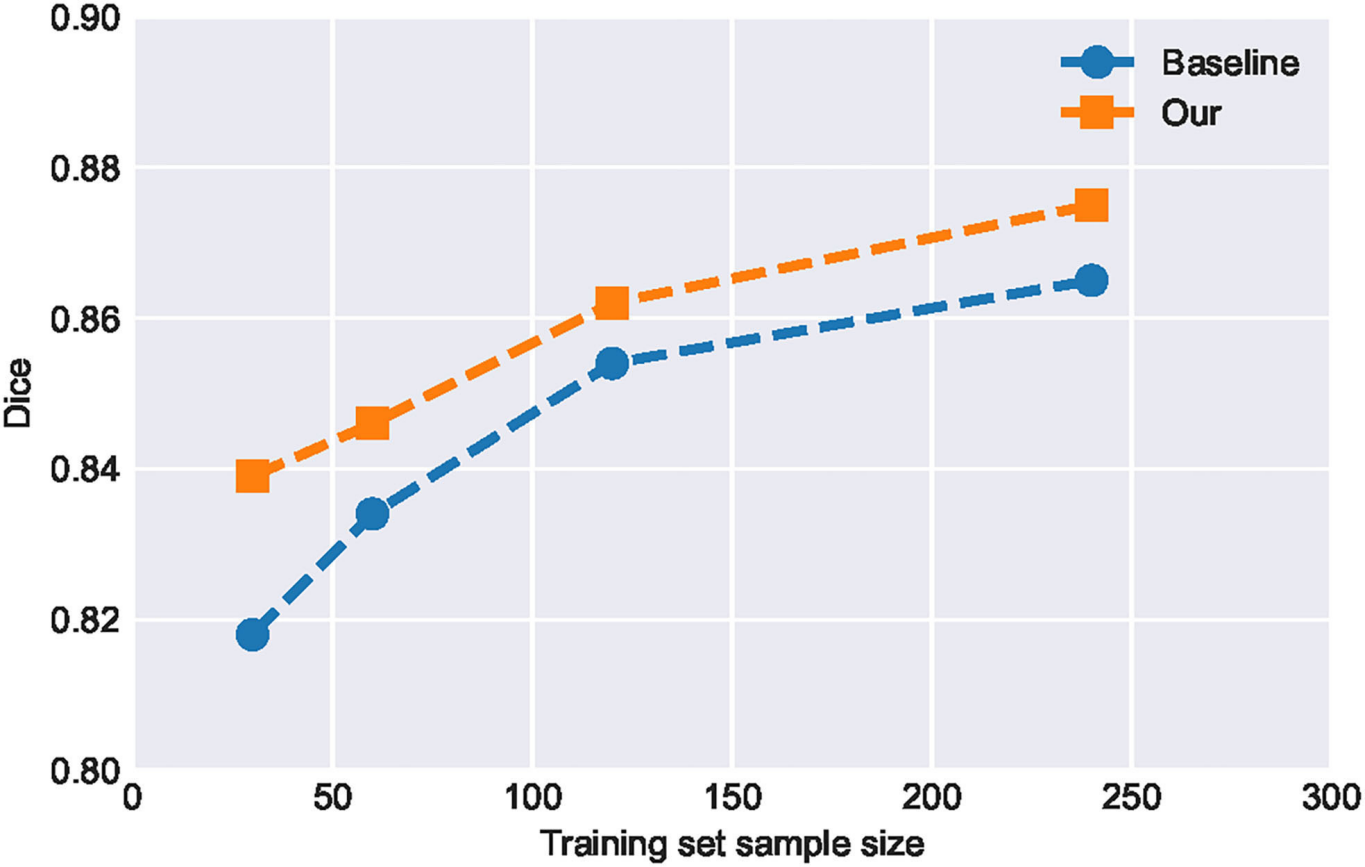
Performance of SA-EN and baseline U-Net under different training set sample sizes. When the training set sample size is small, the effect of SA-EN is greater.

It can be seen from Table 2 that the improvement of SA-EN on the MICCAI dataset is better than the local dataset. The total number of samples in the local dataset is significantly larger than MICCAI dataset. To this end, we analyzed the performance of SA-EN under different training set sample sizes based on the local dataset. First, the testing set was fixed. Then, the sample size of the training was set to 30, 60, 120, and 240, respectively. As shown in Figure 7, the performance of the baseline U-Net and SA-EN both improve with increasing training set sample size. The training set sample size has a significant impact on the model accuracy. Furthermore, the effect of SA-EN gradually reduces with the increase in training set sample size. This result indicates that SA-EN is more effective on small data sets. The detailed results of base models and ensemble are shown in Table 5.

**Table 5:** Performance (Dice, %, higher is better) of SA-EN and baseline U-Net under different training set sample size.

**Task**	**Size**	**Baseline U-Net**	**Base model 1**	**Base model 2**	**Base model 3**	**Base model 4**	**Base model 5**	**Ensemble**
Local dataset	1/10	81.89	82.37	82.43	82.44	82.87	81.82	83.95
	2/10	83.44	83.11	82.98	83.35	83.35	83.45	84.69
	4/10	85.46	85.23	85.37	85.31	85.38	85.47	86.22
	8/10	86.43	86.51	86.43	86.57	86.44	86.33	87.51

The MICCAI dataset includes 60 subjects acquired by three different scanners in three different hospitals (Utrecht, Singapore and Amsterdam, 20 subjects each). To further verify the generalization of SA-EN under fewer subjects, we experiment on three different individual scanners on the MICCAI dataset, respectively. For three different scanners, 20 samples are randomly assigned to the training set (15 samples) and the test set (5 samples). As seen in Table 6, SA-EN achieves a significant Dice gain over the baseline U-Net for all the three different scanners of Amsterdam (↑3.81), Singapore (↑1.94), and Utrecht (↑2.61) segmentation. This result indicates that SA-EN is useful for small datasets. The *p* <0.05 in Table 6 prove that the results are statistically significant.

**Table 6:** Performance (Dice, %, higher is better) of SA-EN on three different scanners in the MICCAI dataset, respectively.

**Scanners**	**Method**	**Base model 1**	**Base model 2**	**Base model 3**	**Base model 4**	**Base model 5**	**Ensemble**	***p*** **-value**
Amsterdam	U-Net	✗	✗	✗	✗	✗	76.16	0.001
	SA-EN	77.03	76.52	76.92	77.16	76.74	79.97	✗
Singapore	U-Net	✗	✗	✗	✗	✗	80.07	0.033
	SA-EN	79.96	79.40	80.12	79.04	79.42	82.01	✗
Utrecht	U-Net	✗	✗	✗	✗	✗	75.46	0.019
	SA-EN	75.53	75.08	75.28	75.55	74.99	78.07	✗

Note that “Baseline” represents the original U-Net. “Base model *k*” represents different base models in EN, and “Ensemble” represents the trained base models are fused. Statistical analysis (p-value) of SA-EN compared with baseline U-Net.

#### 4.3.4. Effect of the ensemble size

Ensemble learning aims at aggregating different base models to boost the segmentation performance. The optimal size of an ensemble, i.e., how many models in the ensemble are needed, remains an open issue and, as in many related EN tasks, a task specific parameter that needs to be optimized. To this end, we set the uncertainty threshold λ from {0.1, 0.2, 0.3, 0.4, 0.5, 0.6, 0.7, 0.8, 0.9, 1.0} and train the corresponding base model respectively. For each model with different sizes of ensembles, the training process was repeated five times.

**Figure 8:**
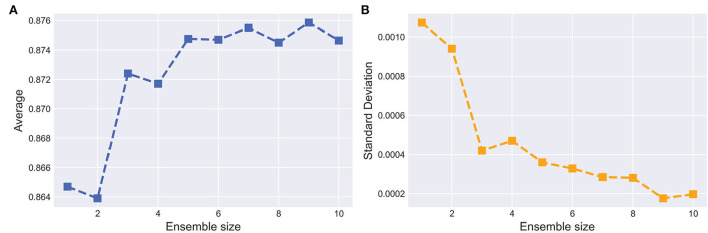
Effect of the ensemble size. **(A,B)** indicate the average and SD of the dice on testing set with respect to ensemble size, respectively. The horizontal axis represents the number of base models in the ensemble.

Figure 8A shows the curves of segmentation performance on dice metrics w.r.t different ensemble sizes. It could be seen that (1) the ensemble with multiple base models outperformed the ensemble with only one base model. (2) when ensemble sizes increased, performance tended to saturate. Figure 8B shows SD of segmentation performance between five repeated trained ensemble models with respect to different ensemble sizes. The variation of segmentation performance was reduced on dice metrics when the ensemble size increased. It demonstrated that the ensemble model not only boosts the segmentation performance but also guarantees a robust segmentation result.

#### 4.3.5. Uncertainty estimation

**Figure 9:**
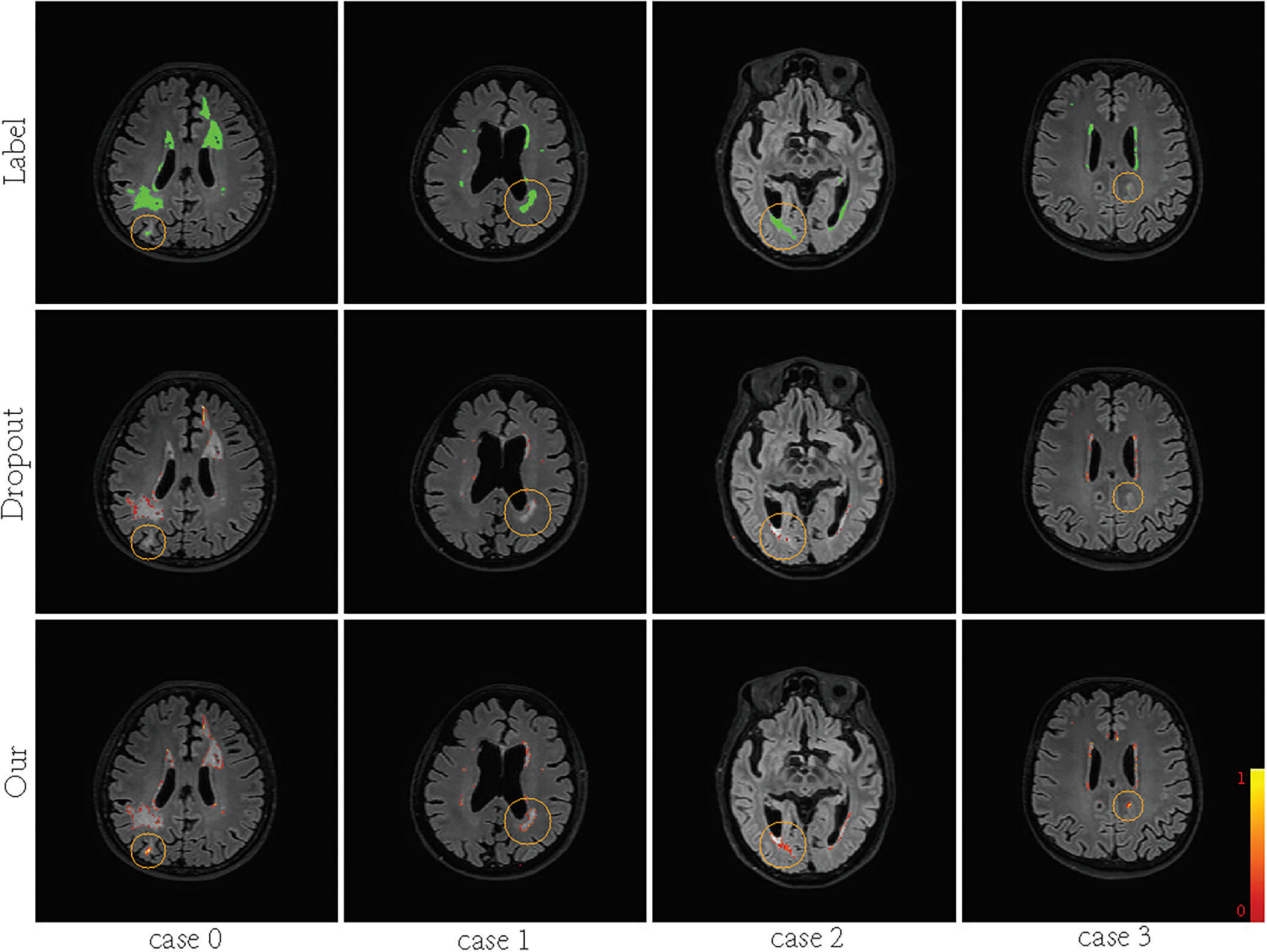
Visualization of the predictive uncertainty computed by Bayesian U-Net and SA-EN. From top row to bottom row: ground truth, the uncertainty computed by Bayesian U-Net and the uncertainty computed by SA-EN. The green areas indicate ground truth. The magnitude of uncertainty corresponds to the color bar in the lower right corner of the plot.

Supervision augmentation can obtain diverse supervision information by estimating the aleatoric uncertainty of annotation. Therefore, SA reflects aleatoric uncertainty to some extent. Then, diverse supervision information is used to train different base models in EN. Ensembles can also be used to estimate epistemic model uncertainty (Lakshminarayanan et al., 2017). Figure 2 shows that SA-EN can get segmentation results and epistemic uncertainty at the same time. Figure 9 shows the epistemic uncertainty of the Monte Carlo dropout method and SA-EN. It can be seen that SA-EN can capture a wider range of prediction uncertainty. The Monte Carlo dropout method can only capture small uncertainty regions. SA-EN uses diverse supervision information to train the base models. This results in a diversity of underlying models after convergence and thus can capture a wide range of uncertainties.

## 5. Discussion

This paper proposes a SA method and combines it with EN to reduce the impact of label noise and ambiguity. SA can obtain diverse supervision information, which is suitable for common single-label scenarios without adding additional data labeling burden. We verify that SA-EN outperforms other state-of-the-art ensemble methods on two white matter hyperintensity segmentation datasets. SA-EN is more effective on small datasets, which is more suitable for medical image segmentation with few annotations. Furthermore, SA-EN can capture two types of uncertainty, aleatoric uncertainty modeled in SA and epistemic uncertainty modeled in EN.

Typically, an ensemble is constructed in two steps. First, a number of base learners are produced. Then, the base learners are combined to use. Generally, to get a good ensemble, the base learners should be as more accurate as possible, and as more diverse as possible. As shown in Figure 4, the difference between these masks is mainly pixels with high uncertainty. These high-uncertainty pixels are mainly distributed at the edges of lesions. The loss function calculated based on these different masks is also diverse. Thus, we can train multiple diversity base networks through these different masks. As shown in Table 2, the segmentation accuracy of these base models is similar. However, the accuracy can be significantly improved after fusing these base models. This also proves that the SA method can provide diverse supervised information. It should be noted that EN based on supervision augmentation is different from the existing ensemble methods based on random initialization, multiple different structures, and sample weighting. We do this by analyzing the annotation quality, which is a new and more efficient implementation. The experiments on two white matter hyperintensity segmentation datasets also show that SA-EN outperforms other state-of-the-art ensemble methods. SA-EN is trained independently and can be easily appended to any existing segmentation tasks and researchers could easily build themselves' segmentation models.

The λ is the uncertainty threshold. As shown in Figure 4, the calculation of the loss function includes more uncertain pixels when λ is set higher. When λ is set low, a large number of uncertain pixels do not participate in the calculation of the loss function. In this paper, after the uncertainty map is normalized, λ is randomly selected from {0.1, 0.2, 0.3, 0.4, 0.5, 0.6, 0.7, 0.8, 0.9, 1.0}. Then, different base models are trained based on the binary masks generated by different λ. Generally, to get a good ensemble, the base learners should be as more accurate as possible, and as more diverse as possible. Table 2 shows that λ has little impact on the performance of the base model. The improved effect after fusion shows that these base models are diverse. SA actually discards some pixels with high uncertainty through λ. Thus, SA is not equivalent to providing under-segmented or over-segmented manual segmentation.

The ensemble methods has *K* times more parameters than a single network. For memory-constrained applications, the ensemble needs to be distilled into a simpler model. In this paper, the base models used in various ensemble methods adopt the U-Net with the same structure. The training time of a single base model on the MICCAI and Local datasets is about 3 and 4 h, respectively. Ensemble methods lead to increased complexity due to multiple base models. But when training base models through SA, they can be trained in parallel. Similarly, they can also be parallel in prediction. However, the complexity of the model parameters is still high. In future work, it would be also interesting to investigate meta-learning and dynamic convolutional networks to solve this problem. These two methods enable a single network to learn multiple objectives simultaneously.

We also analyzed the performance of SA-EN on large and small lesions, respectively. Table 4 shows two methods perform worse in recalling small lesions compared to large lesions. SA-EN significantly improves the segmentation accuracy of large lesions on the two datasets but has little effect on small lesions. We think that the differences between different masks generated by SA are mainly pixels with high uncertainty. As shown in Figure 1, a large number of pixels with high uncertainty are also distributed on the edges of large lesions. Therefore, this may lead to the improvement of our proposed method for large lesions. In the future, we will try to improve the model's ability to segment small lesions, so that the model can significantly improve both large and small lesions.

## Data availability statement

The raw data supporting the conclusions of this article will be made available by the authors, without undue reservation.

## Author contributions

XG and CY conceptualized and designed the study, wrote the first draft of the manuscript, and performed data analysis. LZ manually segmented the WMHs on T2-FLAIR images of the local dataset. YY, LL, and SL performed the experiments, collected and analyzed the data, and revised the manuscript. CG and TM designed the study, gave insight into model improvement, reviewed, and revised the manuscript. All authors contributed to manuscript revision, proofreading, and approved the submitted version.

## Funding

This study is funded by grants from the Innovation Team and Talents Cultivation Program of National Administration of Traditional Chinese Medicine (No: ZYYCXTD-C-202004), Basic Research Foundation of Shenzhen Science and Technology Stable Support Program (GXWD20201230155427003-20200822115709001), the National Key Research and Development Program of China (2021YFC2501202), the National Natural Science Foundation of China (62106113), Shenzhen Longgang District Science and Technology Development Fund Project (LGKCXGZX2020002), the Natural Science Foundation of Jilin Province (No. 20210101273JC), Foundation of Health and Family Planning Commission of Jilin Province (No. 2020J052), Bethune Project of Jilin University (No. 2020B47), and the Science and Technology Achievement Transformation Fund of the First Hospital of Jilin University (No. JDYY2021-A0010).

## Conflict of interest

HL was employed by the company Mindsgo Life Science Company. The remaining authors declare that the research was conducted in the absence of any commercial or financial relationships that could be construed as a potential conflict of interest.

## Publisher's note

All claims expressed in this article are solely those of the authors and do not necessarily represent those of their affiliated organizations, or those of the publisher, the editors and the reviewers. Any product that may be evaluated in this article, or claim that may be made by its manufacturer, is not guaranteed or endorsed by the publisher.

## Appendix

The U-Net used in this paper is shown in Figure 3A. For each patient, the FLAIR and T1 modalities are fed into the U-Net jointly as a two-channel input. It consists of a down-convolutional part that shrinks the spatial dimensions (left side), and up-convolutional part that expands the score maps (right side). The skip connections between down-convolutional and up-convolutional were employed. Here, two convolutional layers are repeatedly employed, each followed by a rectified linear unit (ReLU) and a 2 × 2 max pooling operation with stride 2 for downsampling. At the final layer, a 1 × 1 convolution is used to map vector to two classes. Convolutional layers with 3 × 3 kernel size are heavily used in this paper.

In this paper, the uncertainty estimates follows that of Gal and Ghahramani (2016) which used the dropout at the inference phase. This allowed approximation of the posterior distribution based on the probabilistic softmax output obtained from the stochastic dropout sampling. As in the paper (Hiasa et al., 2019a), the U-Net model was extended as Bayesian U-Net by inserting the dropout layer before each max pooling layer and after each up-convolution layer, as shown in Figure 3B. We call the U-Net extended by MC dropout “Bayesian U-Net.” The dropout layer here is the same as the commonly used regularized dropout (Srivastava et al., 2014). In this paper, the dropout rate of *p* = 0.3 was used. The dropout layer allows to simultaneously optimize the weights to prevent overfitting while modeling the weights distribution. Dropout can be then used at test time to retrieve multiple MC samples by processing the input *X*, *T* times. The resulting outputs can then be averaged to recover a single estimate of the segmentation, and the SD between samples can be taken as an estimate of the uncertainty.
